# Comparison of closed-skill and open-skill exercises on depression symptoms in adults: A systematic review and meta-analysis

**DOI:** 10.1097/MD.0000000000051339

**Published:** 2026-10-30

**Authors:** Yuan Yuan, Lei Shi, Meihua Yuan, Mingrui Shao, Ting Peng, Xiaofa Yuan, Xiupeng Li, Tengxiu Chen, Feng Wang, Aiping Chi, Jing Fan

**Affiliations:** a Physical Education School of Shaanxi Normal University, Xi’an, Shaanxi, China; b Xi’an Jiaotong University, Xi’an, Shaanxi, China; c School of Continuing Education, China University of Geosciences, Beijing, China; d School of Physical Education, Ningxia University, Yinchuan, Ningxia, China; e School of Physical Education, Yancheng Teachers University, Yancheng, Jiangsu, China; f School of Physical Education, Shaanxi University of Technology, Hanzhong, Shaanxi, China; g Shaanxi Vocational and Technical College, Xi’an, Shaanxi, China.

**Keywords:** closed-skill exercise, depression, exercise intervention, open-skill exercise

## Abstract

**Background:**

Depression is a leading cause of disability worldwide, and exercise has been recognized as an important non-pharmacological intervention for improving depressive symptoms. This systematic review and meta-analysis aimed to compare the effects of closed-skill and open-skill exercises on depressive symptoms in adults with depressive disorders or clinically relevant depressive symptoms, and to investigate whether exercise characteristics, including skill type, duration, and frequency, influence intervention effects.

**Methods:**

PubMed, Web of Science, Embase, Scopus, Cochrane Library, and China National Knowledge Infrastructure were searched for randomized controlled trials comparing exercise interventions with non-exercise controls. Exercise interventions were classified as closed-skill or open-skill exercises according to motor skill characteristics. Random-effects meta-analysis was conducted using standardized mean differences (SMDs) with 95% confidence intervals (CIs). Subgroup analyses, meta-regression, funnel plots, Egger’s test, trim-and-fill analysis, leave-one-out sensitivity analyses, and Grading of Recommendations Assessment, Development and Evaluation assessment were performed.

**Results:**

Twenty-one randomized controlled trials were included. Exercise interventions significantly reduced depressive symptoms compared with non-exercise controls (SMD = −0.38, 95% CI = −0.67 to − 0.08), with substantial heterogeneity (*I*^2^ = 72.4%). Closed-skill exercise showed a significant improvement in depressive symptoms (SMD = −0.27, 95% CI = −0.54 to − 0.00), whereas open-skill exercise showed a larger but nonsignificant effect (SMD = −0.83, 95% CI = −2.12 to 0.46). No significant differences were found between closed-skill and open-skill exercises. Meta-regression showed no significant moderating effects of exercise duration or frequency. Publication bias was detected by Egger’s test (*P* = .0038), and trim-and-fill analysis reduced the estimated effect, indicating that the overall findings should be interpreted cautiously. Sensitivity analysis confirmed the stability of results. The certainty of evidence was rated as low according to Grading of Recommendations Assessment, Development and Evaluation.

**Conclusion:**

Exercise interventions can improve depressive symptoms in adults with depressive disorders; however, current evidence does not demonstrate a clear advantage of either closed-skill or open-skill exercise. Closed-skill exercise is supported by a larger evidence base, whereas the potential benefits of open-skill exercise remain uncertain due to limited studies and low-certainty evidence. Further high-quality randomized controlled trials directly comparing different exercise skill characteristics are required.

## 1. Introduction

Depression is a prevalent mental disorder characterized primarily by persistent low mood, loss of interest, and anhedonia, ranking among the leading causes of disability worldwide.^[1]^ Epidemiological data indicate that the prevalence of depression remains high worldwide and is on the rise.^[2,3]^ A meta-analysis of a study covering 30 countries between 1994 and 2014 revealed that the point prevalence of depression in the general population was approximately 12.9%,^[4]^ while during the COVID-19 pandemic, the prevalence of depression in the general population rose to as high as 33.7%.^[5]^

The impact of depression extends across all stages of the life course. Among adolescents, the global point prevalence of depressive symptoms is as high as 34%.^[6]^ University students are also a high-risk group, a meta-analysis of Chinese university students reported a prevalence of 34.7%.^[7]^ Among the adult population, specific groups such as women in the perinatal period^[8]^ and patients with chronic kidney disease^[9]^ also have significantly higher risks of depression. Furthermore, the elderly population faces serious challenges, with global prevalence estimated to range between 19.2 and 28.4%.^[10]^

Although psychotherapy and pharmacotherapy are the first-line treatments for depression, a significant proportion of patients still seek alternative or complementary therapies due to inadequate treatment response, side effects of medication, or personal preference. Physical exercise, as a non-pharmacological intervention, has attracted widespread attention due to its potential to improve mood and alleviate depressive symptoms. However, most previous studies have treated different types of physical exercise as homogeneous interventions, overlooking the potential differences in cognitive and neural mechanisms across various forms of exercise.

In recent years, the field of sports science has begun to distinguish between 2 basic exercise categories based on motor skill characteristics, closed-skill exercises and open-skill exercises. Closed-skill exercises take place in stable, predictable environments, and are typically performed at a pace controlled by the individual, such as running, swimming and weightlifting. By contrast, open-skill exercises take place in dynamic, unpredictable environments and require participants to make rapid decisions and adapt their responses in accordance with constantly changing external cues; examples include basketball, badminton and football.^[11]^

Current evidence suggests that these 2 types of exercise may have different effects on cognitive function, particularly executive function (including inhibitory control, working memory and cognitive flexibility). Several meta-analyses have found that, compared with closed-skill exercises, open-skill exercises may offer more significant benefits in improving executive function.^[12,13]^ Given that depression is often accompanied by executive dysfunction, these differentiated cognitive effects suggest that the effectiveness of interventions for depressive symptoms may also vary depending on the type of sport.

However, there is currently a lack of systematic evidence to elucidate the relative efficacy of open-skill and closed-skill exercise on depressive symptoms. Most studies on the relationship between exercise and depression do not make such a functional distinction between types of exercise, resulting in a lack of clear guidance in clinical practice regarding how to select the most effective type of exercise for patients with depression. This knowledge gap limits the precise application of exercise interventions in the field of mental health. Consequently, this study aims to comprehensively compare the intervention effects of closed-skill and open-skill exercise on depressive symptoms in adults with depression through a systematic review and meta-analysis, with a view to providing an evidence base for the development of more targeted exercise prescriptions.

## 2. Materials and methods

This systematic review and meta-analysis was reported according to Preferred Reporting Items for Systematic Reviews and Meta-Analyses 2020^[14]^ and prospectively registered with PROSPERO (CRD420251232579).

### 2.1. Search strategy

A systematic literature search was conducted in PubMed, Scopus, Web of Science, Embase, Cochrane Library, and China National Knowledge Infrastructure from the inception of each database to August 31, 2026. The search strategy was developed in accordance with the Population, Intervention, Comparison, Outcomes, and Study design framework and combined controlled vocabulary (e.g., MeSH terms) with free-text terms. The search comprised 3 core concept groups: Population, including terms related to depression, depressive disorder, and depressive symptoms; intervention, including terms related to exercise, physical activity, and exercise training; and study design, including terms related to randomized controlled trials and clinical trials. Terms within each concept group were combined using the Boolean operator “OR,” and the 3 concept groups were combined using “AND.” No restrictions were imposed on the publication date during the database searches. In addition, the reference lists of relevant systematic reviews, meta-analyses, and included studies were manually screened to identify potentially eligible studies that were not retrieved through the electronic database searches. Two researchers independently conducted the literature search and cross-checked the retrieved records. Take PubMed as an example, The detailed search strategy for it can be found in Table S1, Supplemental Digital Content 1.

#### 2.1.1. Classification of exercise skill characteristics

Exercise types were classified according to the environment in which the exercise task was performed and the extent to which exercise performance depended on external environmental information. Closed-skill exercises were defined as activities primarily performed in stable and predictable environments, in which task demands and movement conditions remained relatively consistent and participants could largely control the pace and execution of their movements. Examples included running, swimming, cycling, and resistance training. Open-skill exercises were defined as activities performed in dynamic, unpredictable, or continuously changing environments that required participants to continuously perceive external stimuli, make rapid decisions, and adapt their movements to changing environmental conditions. Examples included football, basketball, badminton, and table tennis.^[15]^

For mixed interventions involving both closed-skill and open-skill exercises, classification was based on the dominant training characteristics, including the primary training content, environmental demands, and decision-making requirements specified in the intervention protocol. Studies for which the exercise type could not be clearly determined were excluded from the corresponding skill-type subgroup analysis but were retained in the overall meta-analysis where appropriate. Two researchers independently classified the exercise interventions. Disagreements were resolved through discussion, with consultation with a third researcher when necessary.

### 2.2. Inclusion and exclusion criteria

#### 2.2.1. Inclusion criteria

The inclusion criteria for this systematic review were formulated according to the Population, Intervention, Comparison, Outcomes, and Study design framework. Studies meeting the following criteria were eligible for inclusion: First, population: adults aged ≥ 18 years with a depressive disorder or clinically significant depressive symptoms. Depressive status had to be established using recognized diagnostic criteria or validated depression assessment scales, such as the *Diagnostic and Statistical Manual of Mental Disorders*, the *International Classification of Diseases*, or validated depression symptom scales. Second, intervention: exercise was required to be the primary intervention, including either a single exercise modality or a structured exercise training program. The intervention had to provide sufficient information regarding the exercise prescription, including exercise type, intensity, frequency, duration of each session, and overall intervention duration. Based on motor skill characteristics, exercise interventions were further classified as closed-skill or open-skill exercises. Third, comparison: the comparison group received a non-exercise control condition, including treatment as usual, health education, waiting-list control, or other non-exercise conditions. Fourth, outcomes: studies were required to report at least one validated measure of depressive symptoms or depression severity, including, but not limited to, the Hamilton Depression Rating Scale, Beck Depression Inventory/Beck Depression Inventory-II, Patient Health Questionnaire-9, or Self-Rating Depression Scale. Finally, study design: randomized controlled trials (RCTs).

#### 2.2.2. Exclusion criteria

Studies meeting any of the following criteria were excluded: participants aged under 18, or participants without a depressive disorder or clinically significant depressive symptoms. Studies in which exercise was not the primary intervention, or in which exercise was combined with other interventions and the independent effect of the exercise intervention could not be distinguished. Non-randomized controlled trials, including observational studies, cross-sectional studies, cohort studies, case reports, reviews, meta-analyses and conference abstracts. Studies for which the full text was unavailable, or which lacked the effect size data required to conduct a meta-analysis. Studies in which the type of exercise could not be clearly determined based on the characteristics of the exercise, and which could not be used for relevant subgroup analyses.

### 2.3. Literature screening and data extraction

Using a predesigned, standardized data extraction form, 2 researchers independently conducted the literature screening and data extraction. First, duplicate records were removed, followed by an initial screening of titles and abstracts according to the predefined inclusion and exclusion criteria. The full texts of potentially eligible studies were then retrieved and assessed for eligibility. Disagreements during the screening process were resolved through discussion, with consultation with a third researcher when necessary.

The following information was extracted from the included studies: study characteristics, including first author, year of publication, and country or region; participant characteristics, including sample size, age, sex distribution, depression diagnosis or depressive symptom status, and baseline depression severity; exercise intervention characteristics, including exercise type, motor skill classification (closed-skill or open-skill), exercise intensity, duration per session, exercise frequency, and overall intervention duration; control group characteristics, including the control condition and relevant intervention details; and outcome characteristics, including the depression assessment instrument and baseline and post-intervention data, including means, standard deviations, and sample sizes. For studies with insufficiently reported or missing key data, attempts were made to contact the corresponding authors to obtain the required information.

### 2.4. Assessment of risk of bias

The Cochrane Risk of Bias 2 (RoB 2) tool was used to assess the risk of bias in the included studies. Two researchers independently conducted the risk of bias assessment and evaluated each study in accordance with the RoB 2 guidelines. Any discrepancies were resolved through discussion, with a third researcher consulted when necessary to reach consensus.

RoB 2 assesses 5 domains: bias arising from the randomization process; bias due to deviations from intended interventions; bias due to missing outcome data; bias in measurement of the outcome; and bias in selection of the reported result. Each domain was judged as having low risk of bias, some concerns, or high risk of bias. Based on the assessments across the 5 domains, an overall risk of bias was determined for each study.

### 2.5. Meta-analysis

Statistical analyses were performed using Review Manager 5.4 and R software (4.5.2). As the included studies utilized different assessment tools for depressive symptoms, the standardized mean difference (SMD) was adopted as the effect size, adjusted using Hedges’ *g*, and 95% confidence intervals (95% CI) were calculated.

According to Cohen’s effect size criteria, SMDs of approximately 0.2, 0.5, and 0.8 represent small, medium and large effect sizes, respectively. Given the potential differences among the included studies in terms of population characteristics, exercise intervention programs and study designs, a random-effects model was employed to pool the effects. Heterogeneity across studies was assessed using Cochran’s Q test and the *I*^2^ statistic. *I*^2^ values of approximately 25%, 50%, and 75% represent low, moderate and high levels of heterogeneity, respectively. In accordance with the prespecified research hypotheses, subgroup analyses were conducted according to motor skill type (closed-skill exercise vs open-skill exercise), intervention duration (≤8 weeks vs > 8 weeks) and exercise frequency (≤2 sessions per week vs ≥ 3 sessions per week) to explore potential sources of heterogeneity. Furthermore, meta-regression analysis was employed to further investigate the moderating effects of intervention duration and exercise frequency on effect sizes. Funnel plots and the Egger test were used to assess potential publication bias; for outcome measures where publication bias was suspected, further correction analyses were performed using the trim-and-fill method. The stability of the results was assessed through leave-one-out sensitivity analyses. All statistical tests were conducted using two-tailed tests, with *P* < .05 as the criterion for statistical significance.

### 2.6. Certainty of the evidence

The Grading of Recommendations Assessment, Development and Evaluation (GRADE) approach was used to assess the certainty of evidence for the primary outcome. The GRADE framework initially rates evidence from randomized controlled trials as high certainty and considers 5 factors that may lead to downgrading: risk of bias, inconsistency, indirectness, imprecision, and publication bias. The certainty of evidence was classified into 4 levels: high, moderate, low, and very low.

## 3. Results

### 3.1. Literature search results

The initial database search yielded 5172 records. After removing 2896 duplicate records, 2276 records remained for title and abstract screening, of which 2095 were excluded. Full texts were sought for the remaining 181 reports, although 2 reports could not be retrieved. Of the 179 reports assessed for eligibility, 161 were excluded because they did not meet the predefined eligibility criteria, including ineligible populations, interventions, outcomes, or study designs; non-randomized studies, studies in which the control group received exercise interventions, duplicate publications, and animal studies were also excluded. The initial search therefore resulted in 18 eligible randomized controlled trials.

The search was subsequently updated to August 31, 2026 using the same predefined search strategy and eligibility criteria. The updated search identified 3 additional randomized controlled trials that met the inclusion criteria. Thus, a total of 21 randomized controlled trials were included in the final systematic review and meta-analysis.^[16–36]^ The study identification and selection process is presented in Figure 1.

**Figure 1.**
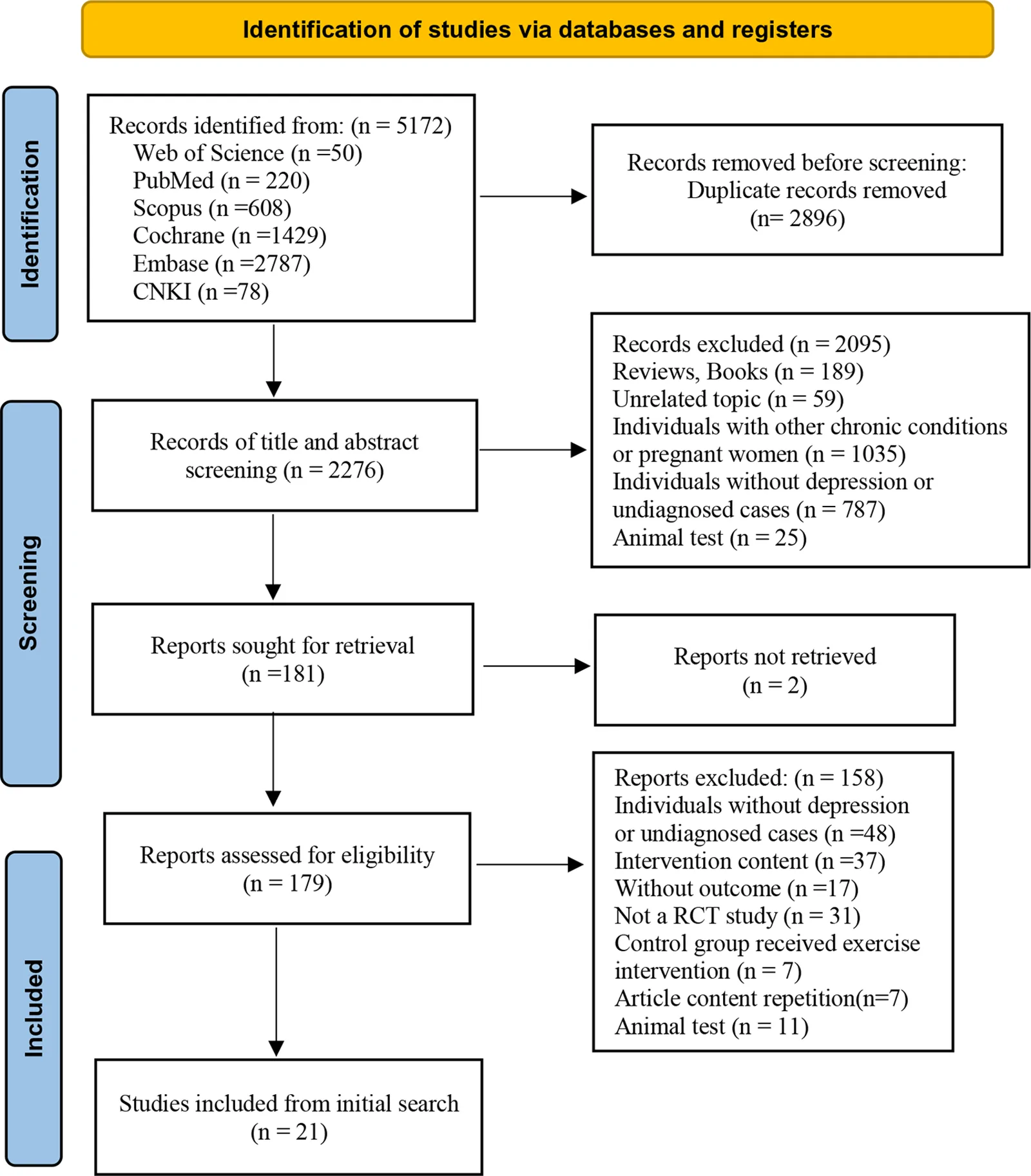
PRISMA flow diagram. CNKI = China National Knowledge Infrastructure, PRISMA = Preferred Reporting Items for Systematic Reviews and Meta-Analyses, RCT = Randomized controlled trial.

### 3.2. Basic characteristics of included studies

This systematic review included 21 randomized controlled trials involving a total of 1005 participants with depressive symptoms or depressive disorders. Based on motor skill characteristics, the included studies were categorized into 2 groups: closed-skill exercise (n = 16) and open-skill exercise (n = 5).

Closed-skill exercise studies primarily involved running, cycling, Nordic walking, Tai Chi, yoga, and resistance training, whereas open-skill exercise studies included table tennis, Taekwondo, dance, and surfing. The studies were conducted mainly in the United States, China, Germany, France, Denmark, the Netherlands, Thailand, Switzerland, South Korea, and Ireland. Participants ranged in age from 18 years to older adults, and intervention durations ranged from 4 to 12 weeks. Exercise frequency ranged from 1 to 5 sessions per week, with individual session durations ranging from 30 to 180 minutes. Depressive symptoms were primarily assessed using validated instruments, including the Beck Depression Inventory/Beck Depression Inventory-II, Hamilton Depression Rating Scale, Korean version of the Geriatric Depression Scale, Montgomery–Åsberg Depression Rating Scale, Patient Health Questionnaire-9, and Self-Rating Depression Scale. Detailed characteristics of the included studies are presented in Tables 1 and 2.

**Table 1:** Basic characteristics of the included studies (closed-skill).

References	Country	Age	Sample size (IG/CG)	Intervention component (IG/CG)	Duration	Intensity	Time	Frequency	Outcome Measures
Krogh et al^[16]^	Denmark	18–60	56/59	Bicycle/TAU	12 wks	65–80%	30 min	3/wk	BDI
Chu et al^[17]^	Tanwan	18–50	13/13	Yoga/walking	12 wks	–	60 min	2/wk	SDI-II
Kabiri Dinani et al^[18]^	China	18–30	9/9	TCC/TAU	8 wks	Moderate	60 min	5/wk	SDS
Lavretsky et al^[19]^	USA	≥60	33/35	TCC/TAU	10 wks	Moderate	120 min	1/wk	HRSD
Kerling et al^[20]^	Germany	18–55	22/20	Bicycle/TAU	6 wks	RPE:≤13	45 min	3/wk	BDI-II
Kruisdijk et al^[21]^	Netherlands	18–65	24/22	Running/TAU	12 wks	60%	60 min	2/wk	HAMD
Legrand et al^[22]^	France	45.3 ± 13.2	14/10	Running/TAU	10 d	Moderate	30 min	1/d	BDI-II
Krause-Sorio et al^[23]^	USA	≥60	26/23	TCC/TAU	12 wks	Moderate	60 min	1/wk	MADRS
Kilpatrick et al^[24]^	USA	>18	21/19	TCC/TAU	12 wks	Moderate	60 min	1/wk	HAMD
Schuver et al^[25]^	USA	20–64	20/20	Yoga/walking	12 wks	Moderate	65 min	2/wk	BDI
Keller-Varady et al^[26]^	Germany	18–60	16/15	Running/TAU	6 wks	Moderate	60–90 min	1/wk	BDI-II
Imboden et al^[27]^	Switzerland	38.9 ± 11.3	29/29	Bicycle/TAU	6 wks	HR60-75%	40–50 min	3/wk	HDRS
Ciccolo et al^[28]^	USA	41.34 ± 12.29	24/21	Weightlifting/TAU	12 wks	RPE10-15	60 min	2/wk	PHQ-9
O’Sullivan et al^[29]^	Ireland	21.8 ± 2.2	27/28	Resistance exercise training	8 wks	70–80% 1RM	40–45 min	2/wk	QIDS
Ginoux et al^[30]^	France	50 ± 12	48/16	Nordic walking	10 wks	65–75% HRmax	60 min	2/wk	BDI-II
Aku et al^[31]^	China	18–30	22/23	Mixed exercise	4 wks	64–75% HRmax	45–50 min	3/wk	PHQ-9

BDI = Beck Depression Inventory, BDI-II = Beck Depression Inventory-II, CG = control group, IG = intervention group, MADRS = Montgomery–Åsberg Depression Rating Scale, PHQ-9 = Patient Health Questionnaire-9, SDS = Self-Rating Depression Scale, TAU = treatment as usual.

**Table 2:** Basic characteristics of the included studies (open-skill).

References	Country	Age	Sample size (IG/CG)	Intervention component (IG/CG)	Period	Intensity	Time	Frequency	Outcome Measures
Zhao et al^[32]^	China	18–23	30/30	Table tennis/TAU	8 wks	65%~75%	30 min	3/wk	BDI-II
Yun et al^[33]^	China	18–25	20/21	Taekwondo/TAU	8 wks	64–76%	30 min	3/wk	BDI-II
Baek et al^[34]^	South Korea	≥65	10/10	Taekwondo//TAU	12 wks	RPE:10 ∼ 13	60 min	3/wk	GDS-K
Haboush et al^[35]^	USA	69.38 ± 5.43	10/12	Dance//TAU	8 wks	–	45 min	1/wk	HRSD
Walter et al^[36]^	USA	18-55	48/48	Surf//TAU	6 wks	Moderate/high	180 min	1/wk	PHQ-9

“–” = not mentioned, BDI-II = Beck Depression Inventory-II, CG = control group, GDS-K = Korean version of the Geriatric Depression Scale, IG = treatment group, PHQ-9 = Patient Health Questionnaire-9, TAU = treatment as usual: including health education, placebo, usual activities, and medication.

### 3.3. Risk of bias

The risk of bias of the 21 included randomized controlled trials was assessed using the RoB 2 tool. Overall, 18 studies were judged to have some concerns, 2 studies were judged to have a low risk of bias, and 1 study was judged to have a high risk of bias.

Across individual domains, 19 studies had a low risk of bias in the randomization process, whereas 16 studies had some concerns regarding deviations from intended interventions. For missing outcome data, 13 studies had a low risk of bias, 7 had some concerns, and 1 had a high risk of bias. The measurement of the outcome was judged to have a low risk of bias in 20 studies, while 19 studies had a low risk of bias in the selection of the reported result. Overall, the most frequent concerns related to deviations from intended interventions and missing outcome data. Detailed assessments are presented in Figures 2 and 3.

**Figure 2.**
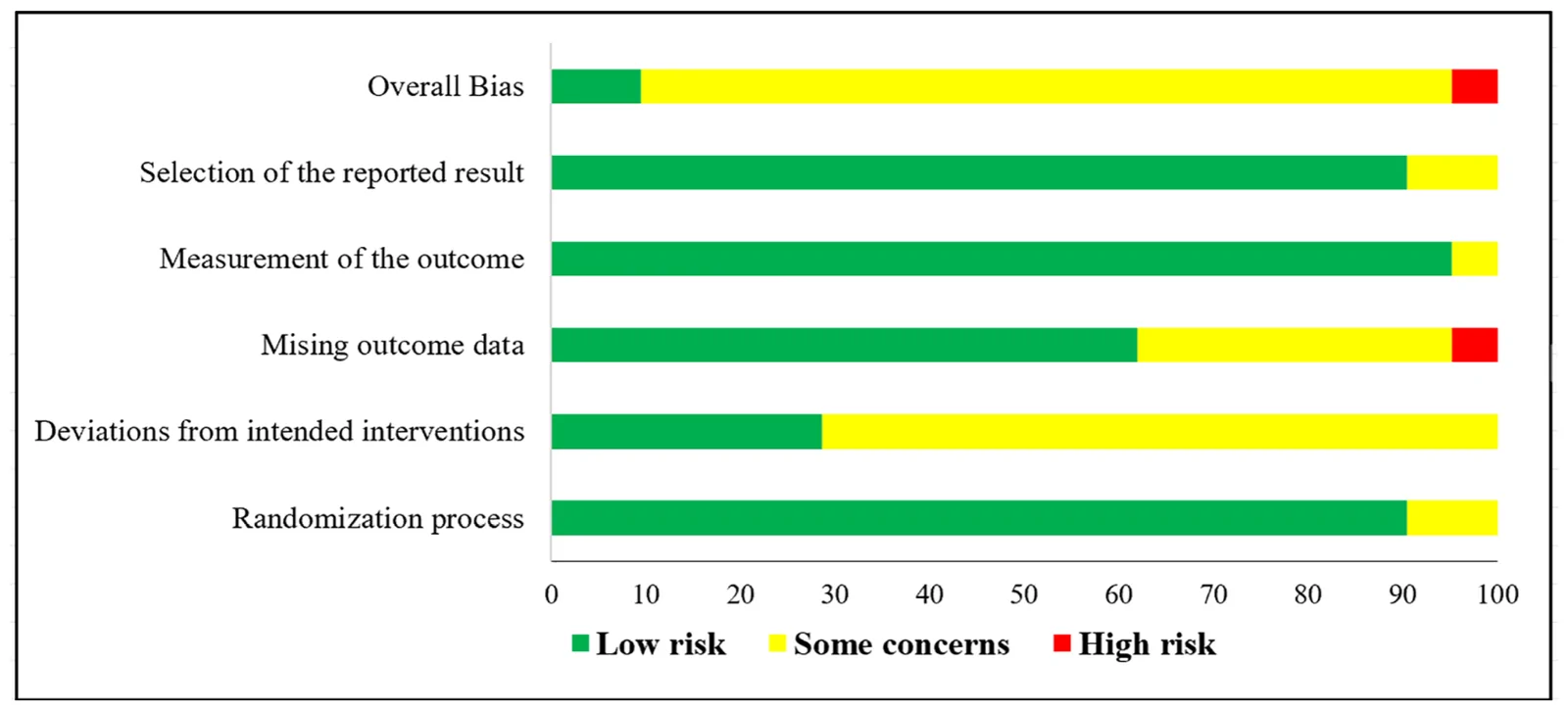
Risk of bias summary.

**Figure 3.**
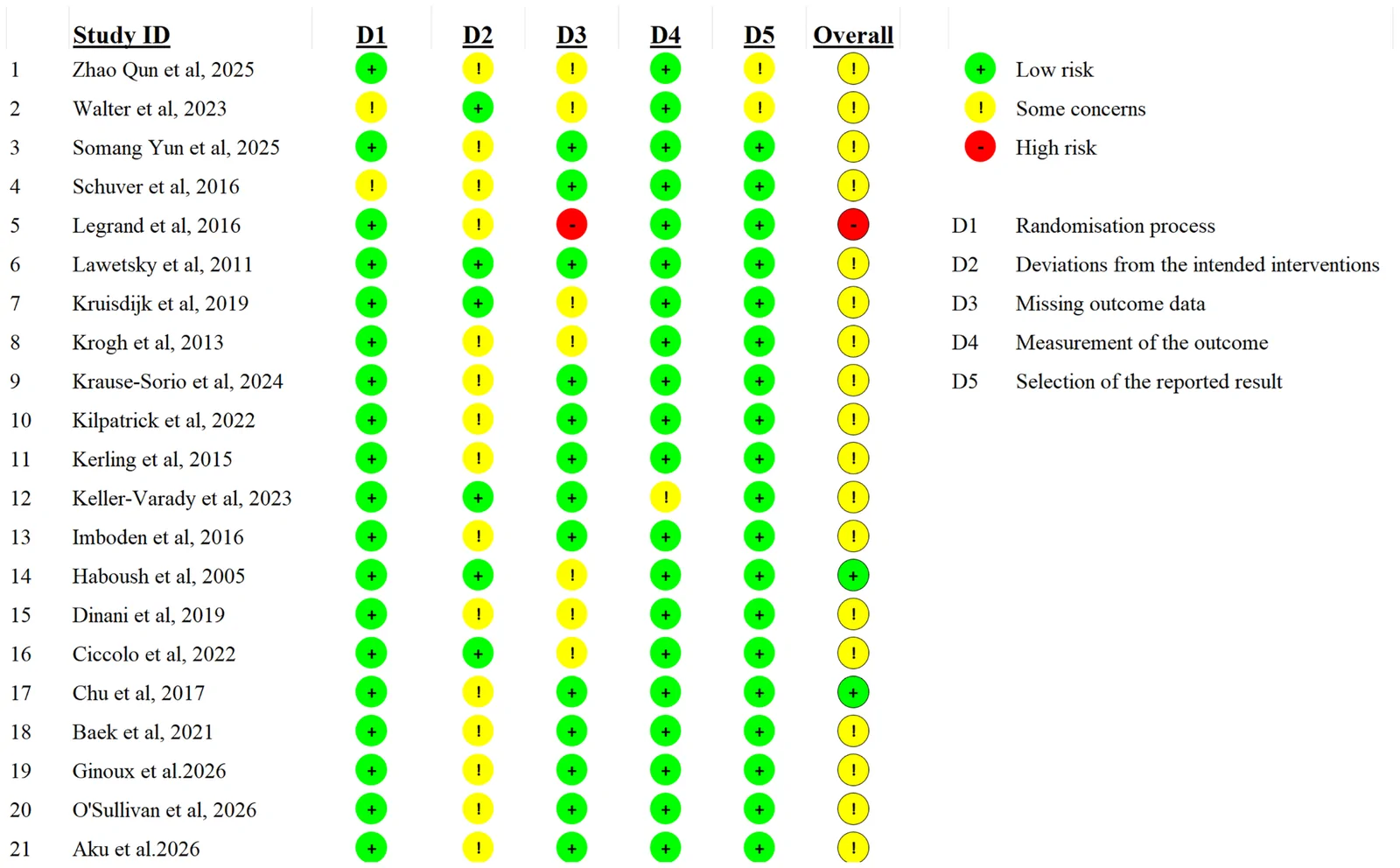
Risk of bias assessment for individual studies.

### 3.4. Meta-analysis results

#### 3.4.1. Overall effect of exercise on depressive symptoms

A random-effects meta-analysis was conducted to evaluate the overall effect of exercise interventions on depressive symptoms. Compared with control conditions, exercise interventions were associated with a significant reduction in depressive symptoms SMD = −0.38, 95% CI (−0.67 to −0.08), *P* = .014. According to Cohen’s criteria, this represents a small-to-moderate effect. However, substantial heterogeneity was observed across the included studies (*I*^2^ = 72.4%), which may reflect differences in participant characteristics, exercise modalities, and intervention protocols. The pooled effect estimate is presented in Figure 4.

**Figure 4.**
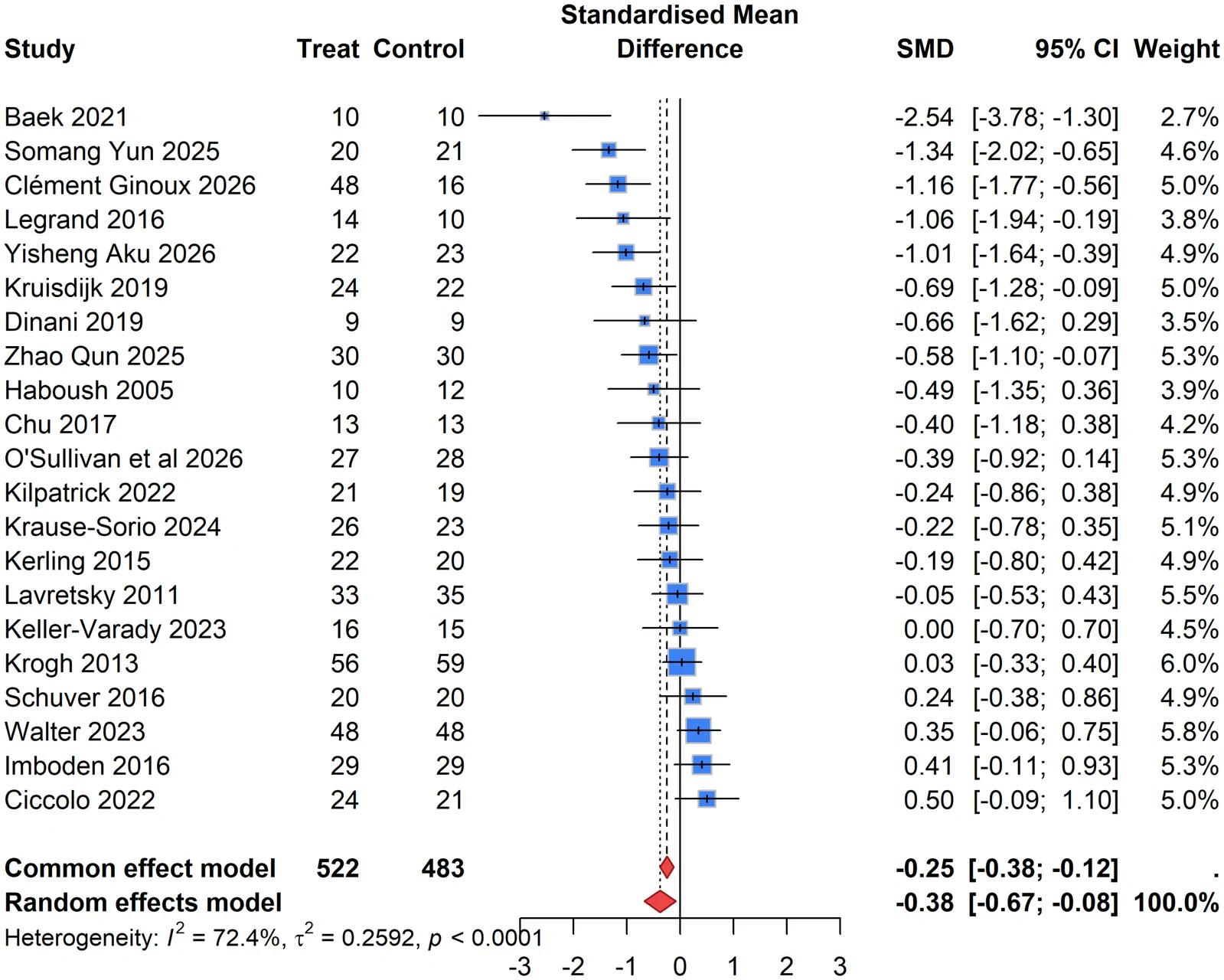
Overall effect forest plot. CI = confidence interval, SMD = standardized mean difference.

#### 3.4.2. Subgroup analysis of the effects of different motor skill types on depressive symptoms

To investigate whether the characteristics of motor skills influence the effectiveness of exercise interventions, this study further conducted a subgroup analysis categorizing the interventions into closed-skill and open-skill exercises. As shown in Figure 5, a total of 16 studies evaluated the impact of closed-skill exercise on depressive symptoms. Results from the random-effects model indicated that closed-skill exercise improved depressive symptoms in adults, with an SMD of − 0.27 and a 95% CI of (−0.54 to −0.00), suggesting that closed-skill exercise has a consistent antidepressant effect. Five studies evaluated open-skill exercise. The results showed that open-skill exercises exhibited a trend towards a larger effect, with an SMD of − 0.83 and a 95% CI of (−2.12 to 0.46); however, as the CI was wide and crossed the null line, this did not reach statistical significance. Subgroup analysis revealed no statistically significant difference between closed-skill exercise and open-skill exercise. The results suggest that, although open-skill exercise may have a greater potential effect, the current evidence is limited and does not yet demonstrate its superiority over closed-skill exercise.

**Figure 5.**
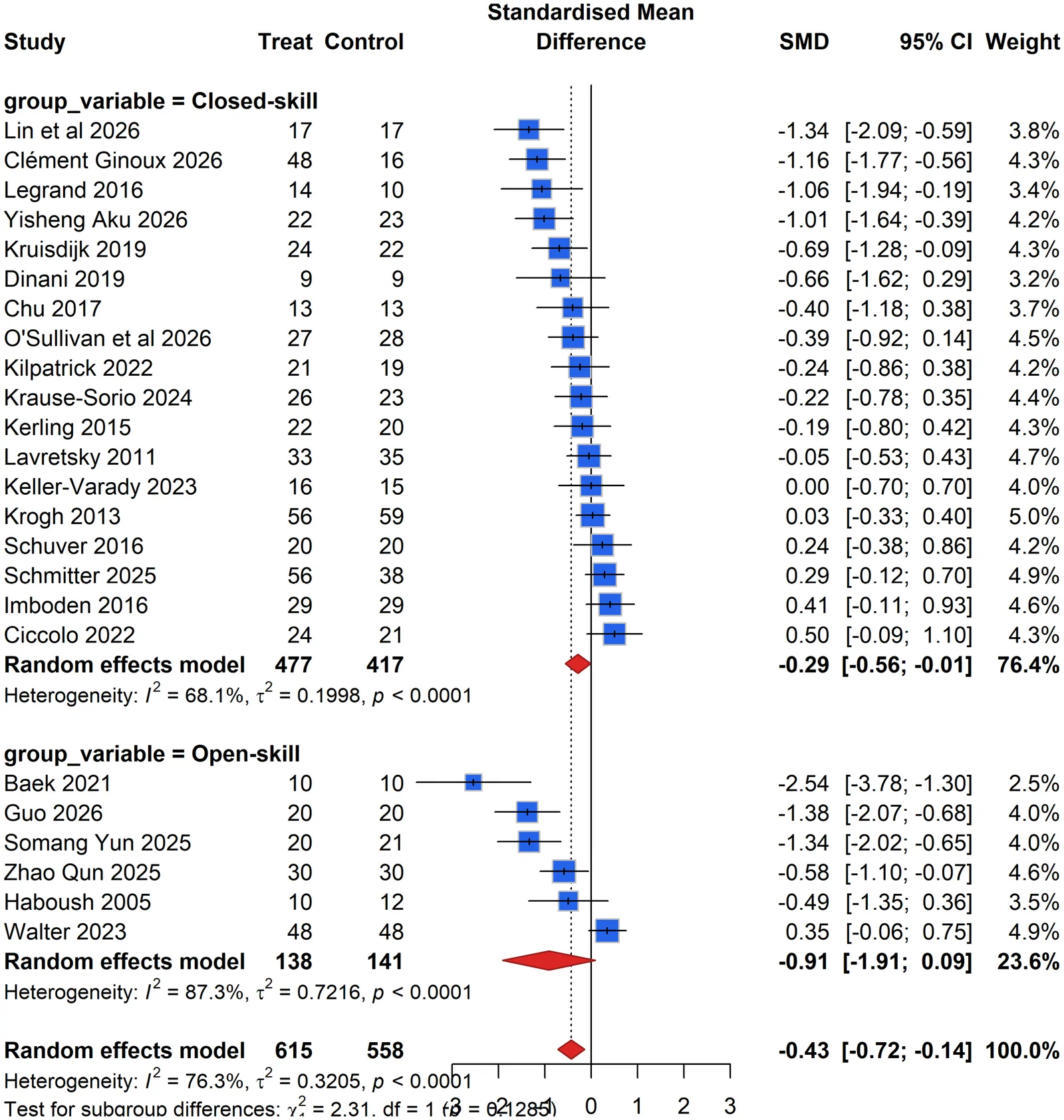
Subgroup analysis of the effects of different motor skills on depressive symptoms. CI = confidence interval, SMD = standardized mean difference.

### 3.5. Exploratory analyses of exercise dose

#### 3.5.1. Subgroup analyses according to intervention duration and exercise frequency

Exploratory subgroup analyses were conducted to examine whether intervention duration and exercise frequency were associated with differences in the effects of exercise on depressive symptoms. As shown in Table 3, interventions lasting ≤ 8 weeks were associated with a statistically significant reduction in depressive symptoms SMD = −0.408, 95% CI (−0.796 to −0.021), *P* = .039, whereas the effect was not statistically significant for interventions lasting > 8 weeks SMD = −0.354, 95% CI (−0.895 to 0.187), *P* = .200. However, the between-subgroup difference was not statistically significant (*P* = .855), indicating no clear evidence that intervention duration modified the treatment effect.

**Table 3:** Subgroup analyses of exercise effects on depressive symptoms by duration and frequency.

Moderator	Subgroup	*k*	SMD	95% CI	*P*	*I* ^2^	*P* for subgroup difference
Duration	≤8 wks	11	−0.408	−0.796, −0.021	.039*	72.7%	.855
	>8 wks	10	−0.354	−0.895, 0.187	.200	74.7%	
Frequency	≤2 sessions/wk	12	−0.190	−0.490, 0.109	.213	61.0%	.110
	≥3 sessions/wk	9	−0.674	−1.299, −0.050	.035*	80.3%	

CI = confidence interval, SMD = standardized mean difference.

* *P* < .05 compared with the control group.

For exercise frequency, interventions delivered ≥ 3 sessions per week were associated with a statistically significant reduction in depressive symptoms SMD = −0.674, 95% CI (−1.299 to −0.050), *P* = .035, whereas the effect was not statistically significant among interventions delivered ≤ 2 sessions per week SMD = −0.190, 95% CI (−0.490 to 0.109), *P* = .213. Nevertheless, the between-subgroup difference was not statistically significant (*P* = .110). Thus, although a significant pooled effect was observed in the higher-frequency subgroup, the available evidence did not demonstrate a statistically significant moderating effect of exercise frequency. Substantial heterogeneity remained within the duration and frequency subgroups (*I*^2^ = 61.0–80.3%). The corresponding forest plot is provided in Figure S1, Supplemental Digital Content 2.

#### 3.5.2. Meta-regression analysis

Meta-regression analyses were further performed to examine whether intervention duration and exercise frequency were linearly associated with the magnitude of the effect on depressive symptoms. No significant association was observed between intervention duration and effect size (β = 0.02, *P* = .684). Exercise frequency showed a negative, but nonsignificant, association with effect size (β = −0.177, *P* = .091), suggesting a potential trend towards greater reductions in depressive symptoms with more frequent exercise; however, this association did not reach statistical significance. The corresponding meta-regression plots are presented in Figures S2 and S3, Supplemental Digital Content 3.

### 3.6. Sensitivity analysis

Leave-one-out sensitivity analyses were conducted to assess the robustness of the pooled effect estimate. Sequential exclusion of each individual study resulted in pooled SMDs ranging from − 0.33 to − 0.42, compared with the overall estimate of − 0.38 (95% CI, −0.67 to − 0.08). Importantly, the direction of the effect remained consistently negative, and the corresponding 95% CIs did not cross the null value after exclusion of any individual study. These findings indicate that the overall effect of exercise on depressive symptoms was robust and was not substantially influenced by any single study. The detailed results of the leave-one-out sensitivity analysis are presented in Figure S4, Supplemental Digital Content 4.

### 3.7. Publication bias

Publication bias was assessed using a contour-enhanced funnel plot (Fig. 6) and Egger’s regression test. The funnel plot showed some asymmetry, and Egger’s test indicated significant asymmetry (*t* = −3.30, df = 19, *P* = .0038), suggesting potential small-study effects. The Duval and Tweedie trim-and-fill analysis (Fig. S5, Supplemental Digital Content 7) identified 7 potentially missing studies, and the adjusted pooled effect was no longer statistically significant (SMD = −0.045, 95% CI − 0.401 to 0.312; *P* = .800), indicating that the overall effect should be interpreted cautiously.

**Figure 6.**
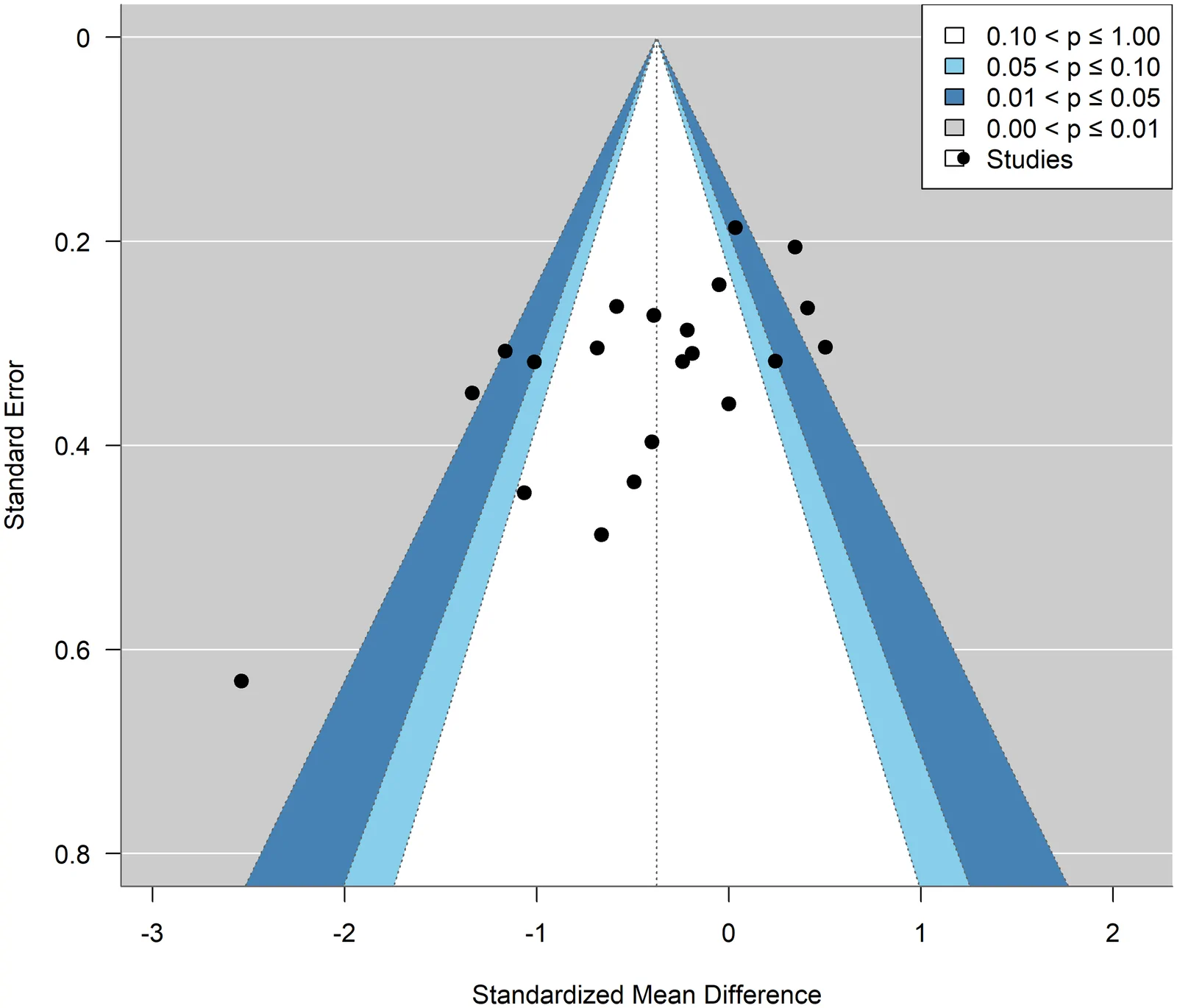
Funnel plot.

### 3.8. Certainty of evidence

The certainty of evidence was assessed using the GRADE framework. The overall certainty was rated as low, with downgrading for risk of bias and inconsistency due to methodological concerns in some studies and substantial heterogeneity (*I*^2^ = 72.4%, *P* < .001). No downgrading was applied for indirectness, imprecision, or publication bias. The pooled evidence from 21 RCTs (n = 1005) indicated that exercise interventions significantly reduced depressive symptoms SMD = −0.38, 95% (CI − 0.67 to −0.08), but the certainty of evidence remained low (Table S2, Supplemental Digital Content 5).

## 4. Discussion

### 4.1. Main findings

This systematic review and meta-analysis aimed to evaluate the overall effects of different exercise skill types on depressive symptoms in adults and to explore the potential influence of exercise dose (i.e., intervention duration and frequency). The overall analysis results confirm the effectiveness of exercise as a non-pharmacological intervention in improving depressive symptoms, which is largely consistent with the conclusions of numerous previous meta-analyses.^[37,38]^ However, substantial heterogeneity was observed across studies (*I*^2^ = 72.4%), indicating considerable variation in the magnitude of benefit. Subgroup analyses suggested a larger effect estimate for open-skill than closed-skill exercise, but the difference between skill types was not statistically significant. Similarly, exercise performed at least 3 times per week and interventions lasting more than 8 weeks showed significant within-subgroup effects, although neither exercise frequency nor intervention duration significantly explained between-study differences. Sensitivity analyses demonstrated that the direction of the pooled effect remained stable when individual studies were sequentially removed. Nevertheless, the significant Egger’s test and the substantial change in the pooled estimate after trim-and-fill analysis indicate that small-study effects may have influenced the observed overall effect. Taken together, the findings support exercise as a potentially beneficial intervention for depressive symptoms, while the comparative advantage of specific exercise characteristics remains uncertain.

The present study provides further support for the beneficial effects of exercise interventions on depressive symptoms in adults, showing a statistically significant small-to-moderate effect (SMD = −0.38). This finding is consistent with the extensive evidence accumulated over the past 2 decades, as numerous systematic reviews and meta-analyses have demonstrated that exercise represents an effective non-pharmacological approach for alleviating depressive symptoms and improving mental health outcomes.^[39,40]^ The mechanisms underlying the beneficial effects of exercise on depressive symptoms are likely multifactorial. From a neurobiological perspective, exercise can enhance the release of neurotransmitters and increase brain-derived neurotrophic factor expression in key brain regions, such as the hippocampus, thereby promoting neuroplasticity and supporting emotional regulation.^[41]^ In addition, regular exercise may modulate neurotransmitter systems involved in mood regulation,^[42]^ contributing to improvements in emotional state and reductions in anxiety symptoms.^[43,44]^ Beyond these biological mechanisms, exercise participation may also enhance self-efficacy and psychological resilience by increasing perceived competence, behavioral control, and positive self-perceptions.^[45]^ Furthermore, participation in group-based exercise or collective physical activities may provide opportunities for social interaction, belongingness, and emotional support, which have been consistently associated with improved psychological well-being and reduced depressive symptoms.^[46,47]^

### 4.2. Exercise type and dose: skill type, duration, and frequency

One of the main contributions of this study is the exploration of whether exercise skill characteristics influence the effects of exercise interventions on depressive symptoms. The subgroup analysis showed that closed-skill exercises (e.g., running, yoga, and resistance training) were associated with a statistically significant improvement in depressive symptoms, which is consistent with previous evidence supporting the mental health benefits of structured exercise interventions.^[48]^ Closed-skill exercises are typically characterized by relatively stable environments, predictable movement patterns, and repetitive practice.^[49]^ These characteristics may contribute to improvements in depressive symptoms through multiple pathways. Regular rhythmic exercise may facilitate autonomic regulation, reduce physiological stress responses, and promote emotional stability. In addition, structured exercise programs may enhance self-efficacy and behavioral activation, which are considered important psychological mechanisms underlying improvements in depressive symptoms. Furthermore, mind–body-based closed-skill activities, such as Tai Chi and yoga, may provide additional psychological benefits by promoting attentional regulation, body awareness, and stress reduction.^[50]^

Interestingly, although open-skill exercises did not reach statistical significance, potentially due to the limited number of available studies and substantial heterogeneity, the estimated effect size was numerically larger than that of closed-skill exercises (SMD = −0.83 vs −0.27). This finding suggests a potential beneficial role of open-skill exercise characteristics that warrants further investigation. Unlike closed-skill exercises, which are performed under relatively stable conditions, open-skill exercises require individuals to continuously perceive environmental changes, make rapid decisions, and adapt movement responses accordingly. Therefore, these activities may provide not only physical stimulation but also increased cognitive demands, involving executive function, attentional control, and sensorimotor processing. Given that depressive symptoms are frequently associated with impairments in cognitive functions, including reduced attentional control and decision-making ability, the additional cognitive engagement required by open-skill exercises may represent a potential pathway through which these activities influence psychological outcomes.^[51,52]^ Moreover, many open-skill exercises involve social interaction and cooperation, which may further contribute to improvements in depressive symptoms by enhancing social connectedness and perceived support.^[53]^ However, these findings should be interpreted cautiously. Although the effect estimate for open-skill exercises was larger than that for closed-skill exercises, the CI was wide, and the number of included studies was limited (n = 5). Furthermore, the difference between exercise skill categories did not reach statistical significance, indicating that current evidence is insufficient to conclude that open-skill exercises are superior to closed-skill exercises for improving depressive symptoms. Therefore, the observed advantage of open-skill exercises should be considered an exploratory finding rather than evidence of a confirmed effect. Future well-designed randomized controlled trials with larger samples and direct comparisons between open- and closed-skill exercises are required to determine whether specific exercise skill characteristics contribute to differential improvements in depressive symptoms.

Beyond exercise skill characteristics, this study further explored whether exercise dose-related factors, including intervention frequency and duration, influenced the effects of exercise interventions on depressive symptoms. Subgroup analyses suggested that exercise frequency may be associated with the magnitude of improvement. Specifically, interventions performed ≥ 3 times per week showed a significant reduction in depressive symptoms SMD = −0.67, 95% CI (−1.30 to −0.05), whereas interventions performed ≤ 2 times per week did not reach statistical significance SMD = −0.19, 95% CI (−0.49 to 0.11). However, the difference between these frequency subgroups was not statistically significant Q = 2.55, *P* = .110, and meta-regression analysis did not identify a significant association between exercise frequency and treatment effects β = −0.177, *P* = .091.

A similar pattern was observed for intervention duration. Although interventions lasting ≤ 8 weeks demonstrated a significant improvement in depressive symptoms SMD = −0.41, 95% CI (−0.80 to −0.02), longer interventions (>8 weeks) showed a favorable but nonsignificant effect SMD = −0.35, 95% CI (−0.90 to 0.19). The difference between duration subgroups was negligible Q = 0.03, *P* = .855, and meta-regression indicated that intervention duration was not a significant predictor of treatment effects β = 0.02, *P* = .684.

These findings highlight the importance of distinguishing between statistical significance within subgroups and evidence of effect modification. Although certain exercise-dose categories showed significant within-group effects, the absence of significant between-group differences and meta-regression associations indicates that current evidence is insufficient to establish exercise frequency or duration as confirmed moderators of improvements in depressive symptoms. The observed patterns may be influenced by other factors, including exercise modality, intensity, participant characteristics, supervision level, and adherence. Therefore, findings regarding exercise dose should be interpreted as exploratory rather than definitive recommendations for an optimal exercise prescription.

Sensitivity analyses indicated that the overall effect was robust, as removal of individual studies did not substantially alter the direction of the pooled estimate. However, publication bias analyses suggested that the magnitude of the observed effect should be interpreted cautiously. Egger’s regression test indicated significant funnel plot asymmetry, and trim-and-fill analysis substantially attenuated the pooled effect estimate, suggesting that small-study effects may have influenced the observed findings. Nevertheless, funnel plot asymmetry may also reflect substantial heterogeneity, differences in sample size, study design, and intervention characteristics rather than publication bias alone. Therefore, the current evidence supports the beneficial direction of exercise on depressive symptoms, although the precise magnitude of the effect remains uncertain.

The GRADE assessment further indicated low certainty of evidence, primarily due to risk of bias, inconsistency, and imprecision. Certainty was particularly low for open-skill exercise because of the limited number of studies and wide CIs. Accordingly, current evidence does not support the superiority of any specific exercise skill type across populations.

### 4.3. Strengths and limitations

This study has several strengths. First, to our knowledge, this is among the first systematic reviews and meta-analyses to specifically compare the effects of open-skill and closed-skill exercises on depressive symptoms in adults, providing a comprehensive evaluation of whether exercise skill characteristics may influence psychological outcomes. Second, this review incorporated a rigorous assessment of methodological quality and certainty of evidence, and further explored potential sources of heterogeneity through subgroup analyses and meta-regression.

Several limitations should also be acknowledged. First, although the overall number of included studies was relatively adequate, the evidence for open-skill exercise was based on only a small number of randomized controlled trials, which limited statistical power and reduced the precision of subgroup estimates. Second, considerable heterogeneity was observed across studies in terms of participant characteristics, exercise modalities, intervention protocols, and outcome assessment tools, which may have influenced the pooled estimates. Third, although sensitivity analyses and publication bias assessments were performed, the presence of potential small-study effects suggests that the magnitude of the observed effects should be interpreted cautiously. Finally, the overall certainty of evidence was rated as low, primarily due to methodological limitations, inconsistency, and imprecision of the available evidence.

### 4.4. Knowledge gaps

As outlined in Section 1 and further discussed above, several knowledge gaps remain in the current literature. No randomized controlled trials have directly compared closed-skill and open-skill exercise in patients with depressive disorder. Second, dose–response relationships – particularly regarding intensity, frequency, duration, and session length – remain poorly defined, especially for open-skill exercise. Third, the open-skill analysis included only 5 studies with substantial heterogeneity (*I*^2^ = 81.4%), limiting the certainty of effect estimates and publication bias assessment. These gaps are partly attributable to the limitations of the present study, Future research should address these gaps to inform clinical decision-making.

### 4.5. Implications and future directions

The findings of this review indicate that exercise interventions, irrespective of skill classification, may provide beneficial effects as an adjunctive strategy for reducing depressive symptoms. However, given the absence of a statistically significant difference between closed-skill and open-skill exercise, the current evidence does not support recommending one exercise type over another. Therefore, exercise prescriptions should be individualized based on patients’ preferences, physical capacity, accessibility, comorbid conditions, and likelihood of long-term adherence. Exercise should be considered a complementary approach rather than a substitute for established pharmacological or psychological treatments. In addition, the limited reporting of exercise intensity and dose parameters in the included studies restricts the development of more precise and evidence-based exercise prescriptions.

Future research should include well-designed randomized controlled trials with larger sample sizes and standardized exercise protocols to determine whether specific skill-related characteristics contribute to greater improvements in depressive symptoms. Detailed reporting of exercise intensity, frequency, and duration is also essential to better understand dose–response relationships. Moreover, future studies may investigate whether changes in executive function, cognitive flexibility, emotional regulation, or social engagement mediate the effects of different exercise skill types on depressive symptoms. Longer-term follow-up is also needed to establish whether the psychological benefits of exercise are maintained after the completion of supervised interventions.

## 5. Conclusion

Exercise interventions can improve depressive symptoms in adults with depressive disorders. however, the current evidence is insufficient to demonstrate a clear advantage of either closed-skill or open-skill exercise. Closed-skill exercise is supported by a larger and more consistent evidence base, whilst open-skill exercise, although showing a larger potential effect, is limited by the small number of studies and uncertainty in the evidence. Future high-quality randomized controlled trials directly comparing different exercise skill characteristics are needed to clarify the role of exercise skill requirements in exercise therapy for depression.

## Author contributions

**Conceptualization:** Yuan Yuan, Tengxiu Chen.

**Data curation:** Yuan Yuan, Tengxiu Chen, Jing Fan.

**Formal analysis:** Yuan Yuan, Tengxiu Chen.

**Funding acquisition:** Aiping Chi, Jing Fan.

**Investigation:** Mingrui Shao, Ting Peng.

**Methodology:** Mingrui Shao, Tengxiu Chen.

**Resources:** Meihua Yuan.

**Software:** Xiaofa Yuan, Xiupeng Li.

**Supervision:** Lei Shi, Ting Peng, Feng Wang, Aiping Chi, Jing Fan.

**Validation:** Xiupeng Li.

**Visualization:** Yuan Yuan, Feng Wang.

**Writing – original draft:** Yuan Yuan.

**Writing – review & editing:** Yuan Yuan.














