# Supplementary material for: Comparison of closed-skill and open-skill exercises on depression symptoms in adults: A systematic review and meta-analysis
Source: Medicine (Baltimore). 2026 Oct 30;105(44):e51339. doi: 10.1097/MD.0000000000051339 (PMC13727200; doi:10.1097/MD.0000000000051339)
Supplement: Supplementary file 1 [file medi-105-e51339-s001.docx]

Table.S1 PubMed Search Strategy

| Steps | Search Strategy |
| --- | --- |
| #1 | (((((depressive disorder[MeSH Terms]) OR (Depression[Title/Abstract])) OR (Major Depressive Disorder[Title/Abstract])) OR (MDD[Title/Abstract])) OR (clinical depression[Title/Abstract])) OR (mood disorder[Title/Abstract]) |
| #2 | (((((((((((((((((((Open Skill[MeSH Terms]) OR (open environment train[Title/Abstract])) OR (variable practice[Title/Abstract])) OR (random practice[Title/Abstract])) OR (basketball[Title/Abstract])) OR (soccer[Title/Abstract])) OR (tennis[Title/Abstract])) OR (martial art[Title/Abstract])) OR (handball[Title/Abstract])) OR (hockey[Title/Abstract])) OR (ice hockey[Title/Abstract])) OR (badminton[Title/Abstract])) OR (table tennis[Title/Abstract])) OR (volleyball[Title/Abstract])) OR (Boxing[Title/Abstract])) OR (judo[Title/Abstract])) OR (wrestling[Title/Abstract])) OR (MMA[Title/Abstract])) OR (fencing[Title/Abstract])) OR (Rugby[Title/Abstract]) |
| #3 | ((((((((((((((((((((Closed training[MeSH Terms]) OR (Closed skills[Title/Abstract])) OR (Fixed-pattern training[Title/Abstract])) OR (Repetitive training[Title/Abstract])) OR (Gymnastics[Title/Abstract])) OR (Track[Title/Abstract] AND field[Title/Abstract])) OR (Swimming[Title/Abstract])) OR (Weightlifting[Title/Abstract])) OR (Stationary cycling[Title/Abstract])) OR (shot put[Title/Abstract])) OR (discus throw[Title/Abstract])) OR (javelin throw[Title/Abstract])) OR (high jump[Title/Abstract])) OR (long jump[Title/Abstract])) OR (running[Title/Abstract])) OR (diving[Title/Abstract])) OR (martial arts[Title/Abstract])) OR (shooting[Title/Abstract])) OR (archery[Title/Abstract])) OR (blocked practice[Title/Abstract])) OR (Athletic[Title/Abstract]) |
| #4 | #2 OR #3 |
| #5 | ( ((RCT[MeSH Terms]) OR (Randomized controlled trial[Title/Abstract])) OR (trial[Title/Abstract])) OR (Clinical trial[Title/Abstract]) |
| #6 | #1 AND #4 AND #5 |
