# Supplementary material for: Comparison of closed-skill and open-skill exercises on depression symptoms in adults: A systematic review and meta-analysis
Source: Medicine (Baltimore). 2026 Oct 30;105(44):e51339. doi: 10.1097/MD.0000000000051339 (PMC13727200; doi:10.1097/MD.0000000000051339)
Supplement: Supplementary file 2 [file medi-105-e51339-s005.docx]

Table S2. Summary of findings and certainty of evidence

| Comparison | No.of studies(n) | Pooled effect (SMD, 95%CI) | Heterogeneity(I^2^) | GRADE certainty | Reasons for ratingdown |
| --- | --- | --- | --- | --- | --- |
| Exercise vs control | K=21, n=1005 | -0.38 [-0.67, -0.08] | 72.4% | Low ⊕⊕⊖⊖ | a, b |
| Closed-skill exercise vs control | K=16, n=766 | -0.27 [-0.54, -0.00] | 61.6% | Low ⊕⊕⊖⊖ | a, b |
| Open-skill exercise vs control | K=5, n=239 | -0.83 [-2.12, 0.46] | 87.6% | Very Low ⊕⊖⊖⊖ | a, b, c |

GRADE Working Group grades of evidence:

⊕⊕⊕⊕ High certainty: further research is very unlikely to change confidence in the estimate.

⊕⊕⊕⊖ Moderate certainty: further research may have an important impact on confidence in the estimate.

⊕⊕⊖⊖ Low certainty: confidence in the effect estimate is limited; the true effect may be substantially different from the estimated effect.

⊕⊖⊖⊖ Very low certainty: there is very little confidence in the effect estimate.

Downgrade criteria:

a Risk of bias: downgraded due to concerns regarding deviations from intended interventions and missing outcome data in several randomized controlled trials.

b Inconsistency: downgraded due to substantial heterogeneity among studies.

c Imprecision: downgraded due to wide confidence intervals and limited sample sizes.

d Indirectness: downgraded because evidence was derived from heterogeneous exercise modalities and populations rather than direct comparisons between open- and closed-skill exercises.

e Publication bias: downgraded due to significant funnel plot asymmetry and sensitivity of pooled estimates to trim-and-fill adjustment.
